# Supplementary figures and images for: Measuring the diffusion of innovations with paragraph vector topic models
Source: PLoS One. 2020 Jan 22;15(1):e0226685. doi: 10.1371/journal.pone.0226685 (PMC6976149; doi:10.1371/journal.pone.0226685)

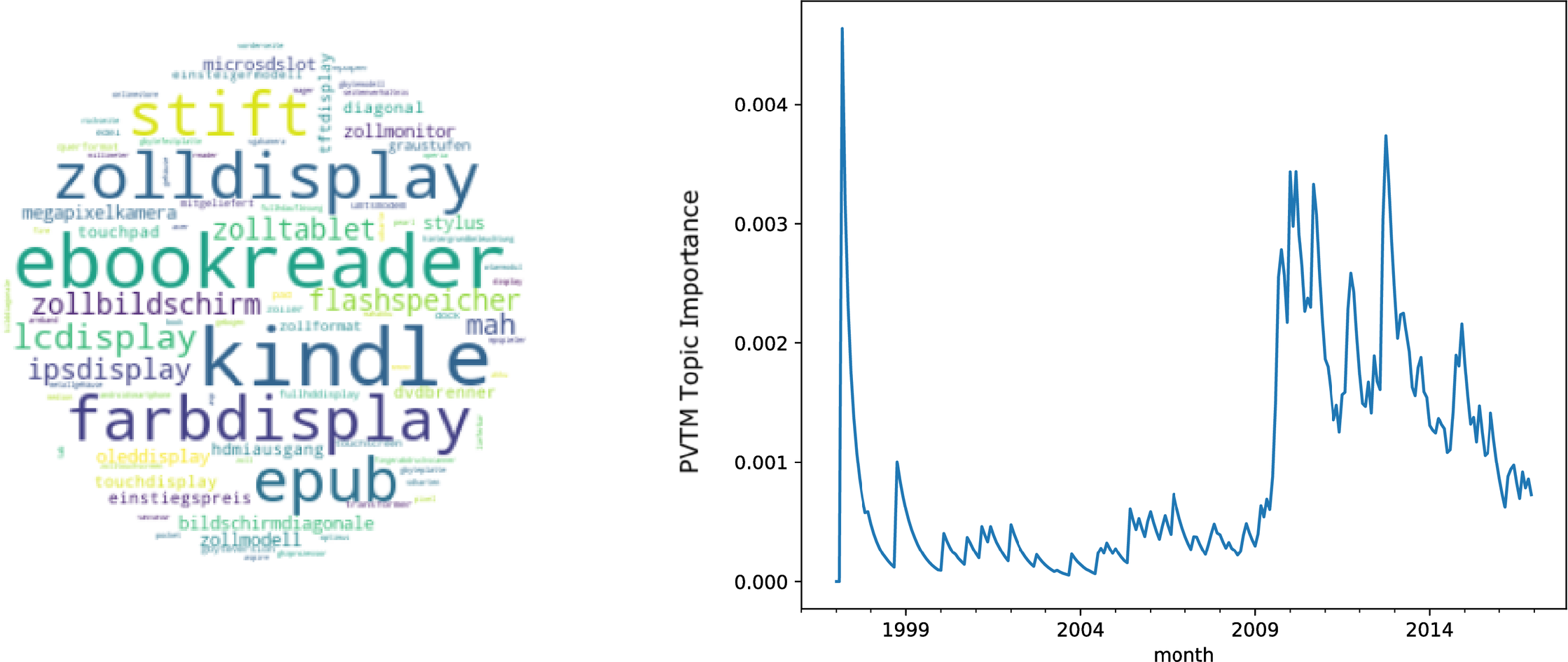

Supplement: S1 Fig — A: Most relevant words. B: Monthly observations of the topic importance over time, smoothed using a 12-month exponentially weighted moving average. (TIF) [file pone.0226685.s001.tif]

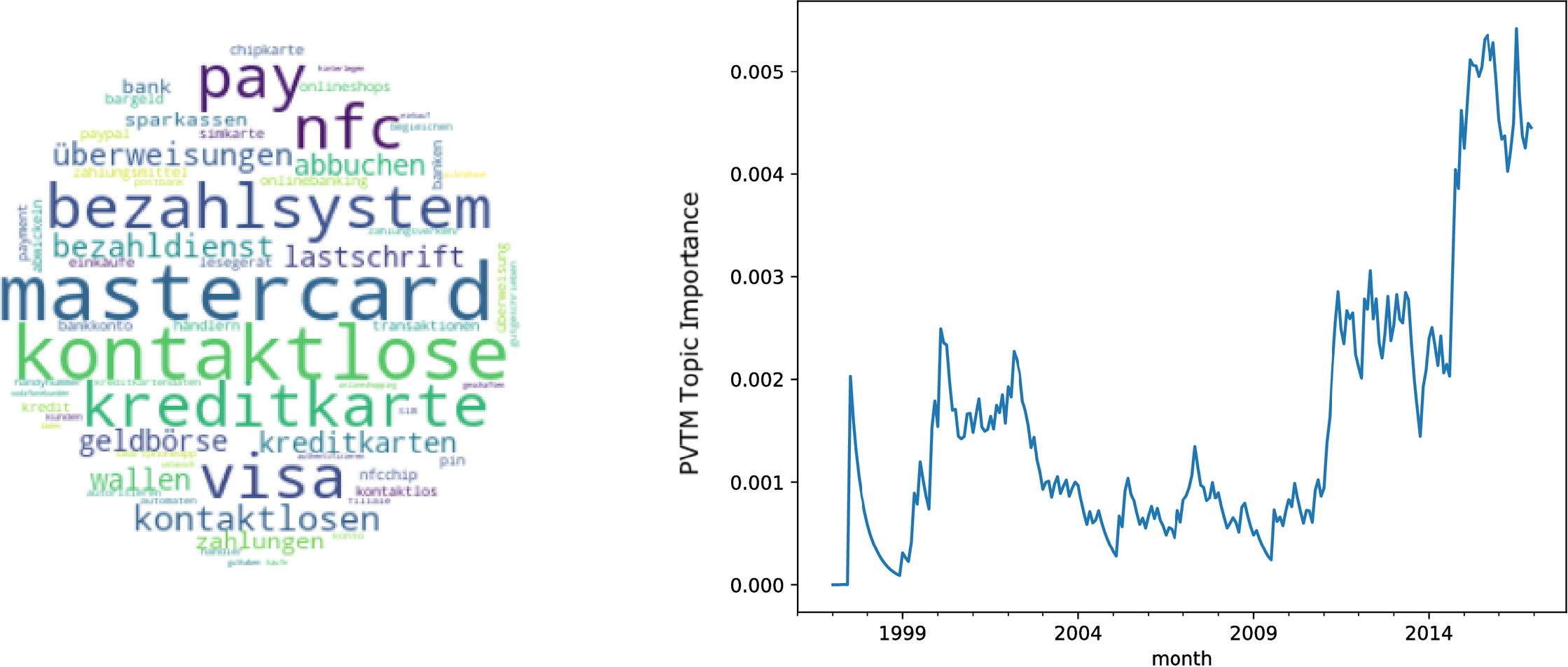

Supplement: S2 Fig — A: Most relevant words. B: Monthly observations of the topic importance over time, smoothed using a 12-month exponentially weighted moving average. (TIF) [file pone.0226685.s002.tif]

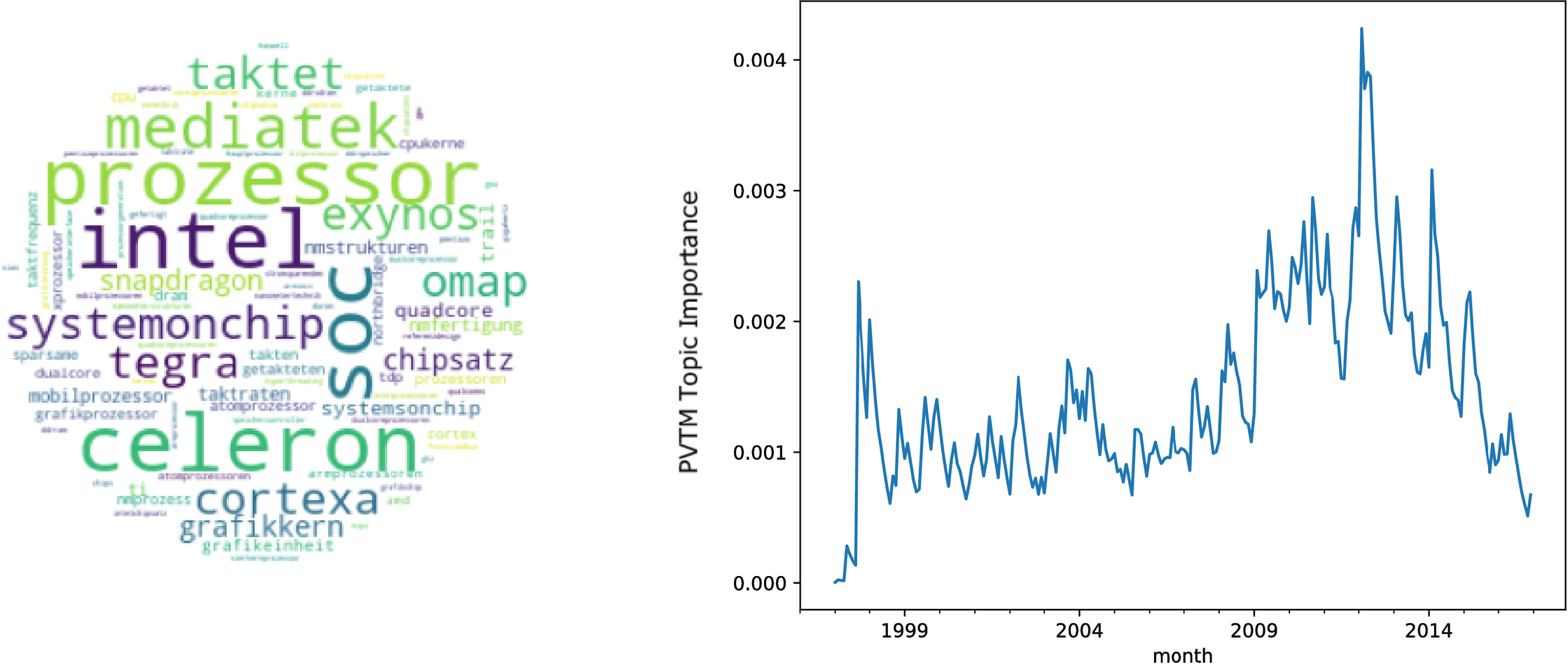

Supplement: S3 Fig — A: Most relevant words. B: Monthly observations of the topic importance over time, smoothed using a 12-month exponentially weighted moving average. (TIF) [file pone.0226685.s003.tif]

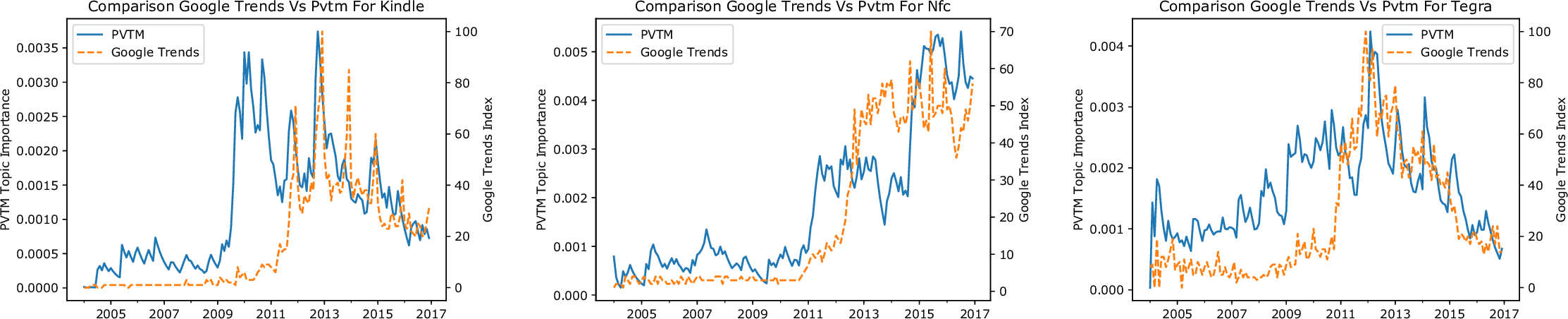

Supplement: S4 Fig — A: kindle topic. B: nfc topic. C: tegra topic. Monthly PVTM observations were smoothed using a 12-month exponentially weighted moving average. (TIF) [file pone.0226685.s004.tif]

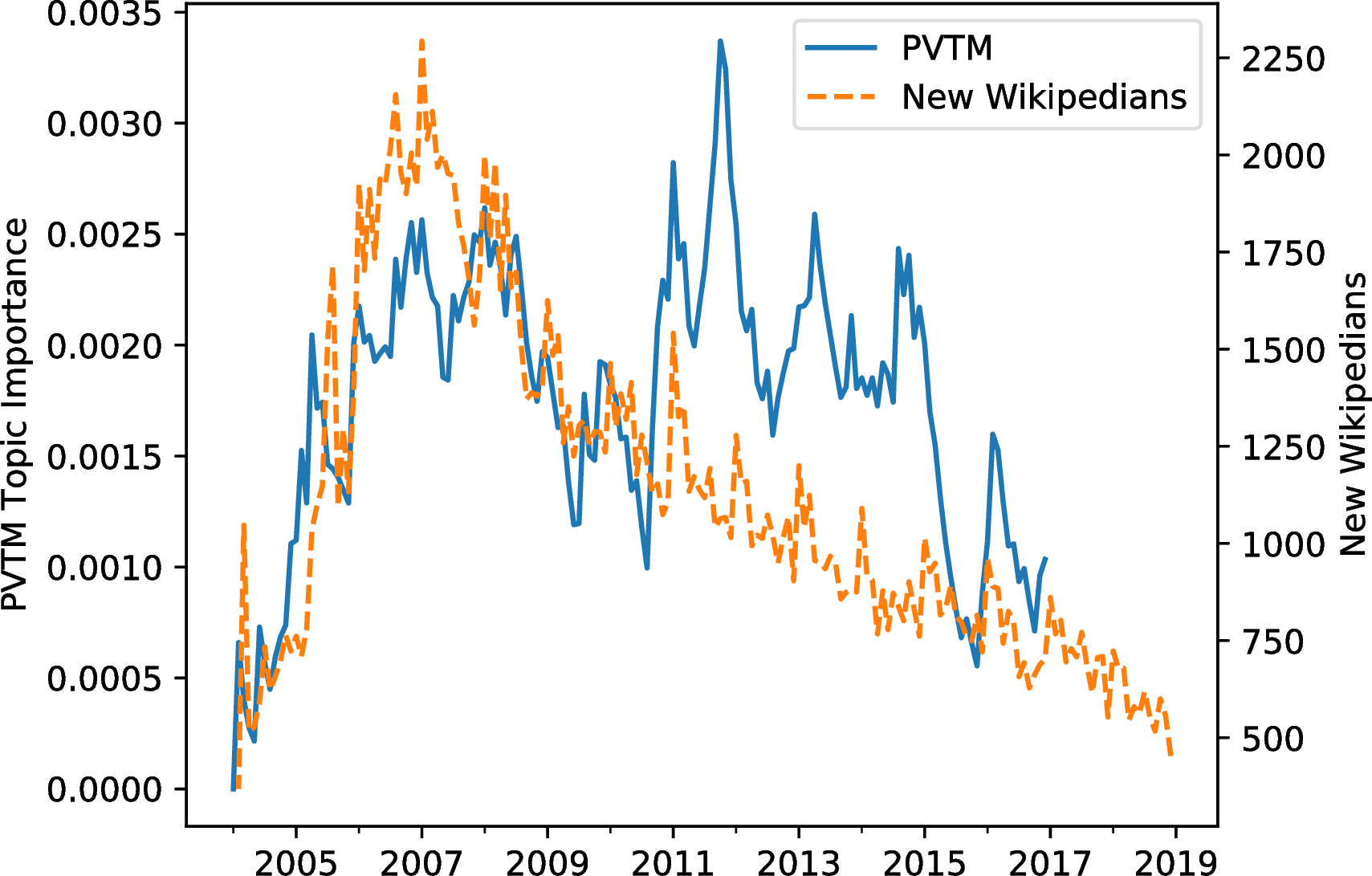

Supplement: S5 Fig — Monthly observations of the topic importance over time, smoothed using a 12-month exponentially weighted moving average, compared against the number of new wikipedians in Germany. (TIF) [file pone.0226685.s005.tif]
