# Supplementary material for: Measuring the diffusion of innovations with paragraph vector topic models
Source: PLoS One. 2020 Jan 22;15(1):e0226685. doi: 10.1371/journal.pone.0226685 (PMC6976149; doi:10.1371/journal.pone.0226685)
Supplement: Attachment — Submitted filename: Referee_Report.pdf [file pone.0226685.s006.pdf]

Measuring the diffusion of innovations with Paragraph Vector top ic models

Referee report

PLOS ONE - PONE-D-19-15216

This paper uses PVTM to look at the diffusion of innovation using a corpus of articles from a German newspaper. The dynamics of each topic is then shown to carry some information which are different from what is found in the more standard measure using Google Trends. The paper is very well written and pedagogic and the analysis is done carefully. I mostly have minor comments.

- Given the trans-disciplinary nature of the review, I believe it would be very insightful to have a short discussion of recent contributions regarding NLP applied to innovation related corpus (patents, scientific articles, newspaper, website etc...) and a brief overview of the evolution of the field (including some of your own works).
- In particular, how does your method compare to alternative (and older) classification methods (just to name a few examples: Nichols, 2014 using LDA, Chavalarias and Cointet, 2013, Bergeaud et al., 2017 using clustering and tdf/idf approaches and other older work e.g. Yoon and Park (2004) and Choi and Hwang, 2014) or more recent contribution (Abood and Feltenberger, 2018, Helmers et al., 2019)
- The number of words per article seems very low (278 on average). Is that specific to the German language (with whom I am unfortunately not familiar) or is it specific to the type of news you are considering?
- You picked 65% and 0.5% as thresholds in the data prepossessing. Could these thresholds be optimized in order to maximize one feature of your network (modularity, number of topics etc...)? More generally, can you discuss the sensitivity of your approach to adjusting these numbers?
- It would be super nice if you could validate the fact that your outcome is a good measure of the diffusion of innovation using an alternative dataset that is aiming at directly measuring such a diffusion. I am aware this is an impossible task for every topic, but perhaps you could find one or two nice examples. An example of such database is Comin and Hobijn (2009) but the world bank and the OECD also entertain cross country measures of innovation diffusion.
- Why do you think your indicator carry additional information than the Google Trends? My guess from the graph you shown is that the google trends reflects the intensive margin of innovation while the topic based measure you use reflects the

density this diffusion (people continue to search for information regarding tablets even when this technology is fully diffused, but newspaper articles won't talk about it that much).

- I find it interesting that contrary to the google trend measure that show spike corresponding to the release of new vintages, your measure is much smoother.
- There are typos at the very end of Section 1

I would like to state again that I really liked the paper as it is, my main point regards the literature review that could be extended given the dynamics of this field. But most of my other comments are minor and I would be happy to just see a discussion about them.

References

Abood, Aaron and Dave Feltenberger, "Automated patent landscaping," *Artificial Intelligence and Law*, 2018, *26* (2), 103–125.

Bergeaud, Antonin, Yoann Potiron, and Juste Raimbault, "Classifying patents based on their semantic content," *PloS one*, 2017, *12* (4), e0176310.

Chavalarias, David and Jean-Philippe Cointet, "Phylomemetic patterns in science evolutionthe rise and fall of scientific fields," *PloS one*, 2013, *8* (2), e54847.

Choi, Jinho and Yong-Sik Hwang, "Patent keyword network analysis for improving technology development efficiency," *Technological Forecasting and Social Change*, 2014, *83*, 170–182.

Comin, Diego A and Bart Hobijn, "The CHAT Dataset," Working Paper 15319, National Bureau of Economic Research September 2009.

Helmers, Lea, Franziska Horn, Franziska Biegler, Tim Oppermann, and Klaus-Robert Müller, "Automating the search for a patents prior art with a full text similarity search," *PloS one*, 2019, *14* (3), e0212103.

Nichols, Leah G, "A topic model approach to measuring interdisciplinarity at the National Science Foundation," *Scientometrics*, 2014, *100* (3), 741–754.

Yoon, Byungun and Yongtae Park, "A text-mining-based patent network: Analytical tool for high-technology trend," *The Journal of High Technology Management Research*, 2004, *15* (1), 37–50.
