# Supplementary material for: Measuring the diffusion of innovations with paragraph vector topic models
Source: PLoS One. 2020 Jan 22;15(1):e0226685. doi: 10.1371/journal.pone.0226685 (PMC6976149; doi:10.1371/journal.pone.0226685)
Supplement: Attachment — Submitted filename: Response to Reviewers.pdf [file pone.0226685.s007.pdf]

Response to the Editor

1. *Please ensure that your manuscript meets PLOS ONE's style requirements, including those for file naming.*

   We carefully read the style requirements and did our best to satisfy all requirements.

2. *Is the manuscript technically sound, and do the data support the conclusions?*

   The manuscript must describe a technically sound piece of scientific research with data that supports the conclusions. Experiments must have been conducted rigorously, with appropriate controls, replication, and sample sizes. The conclusions must be drawn appropriately based on the data presented.

   Reviewer 1: Partly

   Reviewer 2: Yes

   Reviewer 3: Yes

   Reviewer 4: Yes

   We hope that the revised version of the paper will fully satisfy the reviewers.

3. *Has the statistical analysis been performed appropriately and rigorously?*

   Reviewer 1: Yes

   Reviewer 2: Yes

   Reviewer 3: Yes

   Reviewer 4: Yes

   -

4. *Have the authors made all data underlying the findings in their manuscript fully available?*

The PLOS Data policy requires authors to make all data underlying the findings described in their manuscript fully available without restriction, with rare exception (please refer to the Data Availability Statement in the manuscript PDF file). The data should be provided as part of the manuscript or its supporting information, or deposited to a public repository. For example, in addition to summary statistics, the data points behind means, medians and variance measures should be available. If there are restrictions on publicly sharing datae.g. participant privacy or use of data from a third partythose must be specified.

Reviewer 1: No

Reviewer 2: Yes

Reviewer 3: No

Reviewer 4: No

We have uploaded a script to github gist (`https://gist.github.com/davidlenz/2f14b28ead67ee1522c2d5fcbf76a45e`) with which the texts can be scraped from the website. As we do have explicit permission to work with the data but are not allowed to share the data, this is unfortunately the farthest we can go in providing access to our data source.

5. *Is the manuscript presented in an intelligible fashion and written in standard English? PLOS ONE does not copyedit accepted manuscripts, so the language in submitted articles must be clear, correct, and unambiguous. Any typographical or grammatical errors should be corrected at revision, so please note any specific errors here.*

   Reviewer 1: No

   Reviewer 2: Yes

   Reviewer 3: Yes

   Reviewer 4: Yes

   The revised paper has been carefully proof read. We also used a paper check service to have the paper proof read.

Response to Reviewer 1

What the paper does: This article proposes to use newly developed Paragraph Vector algorithms together with a Gaussian mixture model to estimate technology related news topics from a large news corpus and measuring their diffusion across time. The derived innovation topics are shown to Granger cause (predict) Google trends indices with matching search terms. General comments: I find the paper interesting, but with large room for improvement. The idea, to use news articles and their underlying topics to measure diffusion of innovation seems sensible and novel. The approach, using paragraph vectors coupled with a Gaussian mixture models is creative and well placed. Finally, most of the analysis seems well conducted and the presentation is reasonably clear. However, I have two main objections with the paper: 1) First, I find the result section rather disappointing. It seems to me that they only focus on three handpicked dimensions of this very high dimensional problem. I would like to see a much richer presentation of the results from their analysis in this respect. E.g., results for a wide range of innovation terms. Maybe using actual patent data registers might be useful here? 2) Second, as the article is written now, it is somewhat unclear what the methodological contribution is. Is it the how the authors use the paragraph embedding technology together with the Gaussian clustering algorithm, or is it something more fundamental. This should be clarified better.

We are indebted to the referee for the careful reading of our review paper and his/her overall positive assessment. We will describe in detail below how we reacted to the specific comments made while preparing our revised version.

1. *First, I find the result section rather disappointing. It seems to me that they only focus on three handpicked dimensions of this very high dimensional problem. I would like to see a much richer presentation of the results from their analysis in this respect. E.g., results for a wide range of innovation terms. Maybe using actual patent data registers might be useful here?*

   We thank the referee for his comments. First, we have to apologize that we uploaded the wrong images for Figures 3 - 5, which were meant to show a wordcloud with timeline instead of the timeline only. We have

fixed this error and uploaded the correct images, which now provide more information about the content of the topics by means of these wordclouds. Furthermore, we extended the presentation of results, and the paper now includes an Appendix with three more examples, namely for the topics "kindle", "nfc" and "tegra". We agree with the reviewer that a comparison with patent registers could provide additional insights. However, given that the outcome of our analysis are innovation topics such as tablet, it is not straightforward to select corresponding entries in patent databases. Therefore, such an analysis is beyond the scope of our paper, which has its main focus on introducing the PVTM topic model (compare answer to next comment) and demonstrating the applicability of this topic model as means to measure the diffusion of innovations. Nevertheless, we will keep the idea of a comparison with patent data for future research.

2. *Second, as the article is written now, it is somewhat unclear what the methodological contribution is. Is it the how the authors use the paragraph embedding technology together with the Gaussian clustering algorithm, or is it something more fundamental. This should be clarified better.*

   We thank the referee for pointing out that the paper is not clear enough about its methodological contributions. Therefore, we added more details regarding the methodological contributions right at the start of Section 1 (Paragraph Topic Vector Modeling).

3. *I did not really understand the last sentence of the abstract*

   We thank the reviewer for this indication. We agree that the last part of the abstract was not well structured. Thus, we rephrased it by changing the order of the last two sentences in the abstract.

4. *ii) The authors should cite and relate to the article by Measuring Technological Innovation over the Long Run, by Bryan Kelly, Dimitris Papanikolaou, Amit Seru, Matt Taddy (2018). How do your paper differ, how do you contribute etc. ?*

We are indebted to the referee for pointing out this relevant reference, which we had missed unfortunately in our literature search. We carefully read the paper and included a reference to it into the extended literature review, also discussing the differences between our approach and the one presented in that paper.

5. *iii) Line17 to 19: These are typical LDA assumptions that need not be general for all other types of existing topic models. Should be clarified.*

   We agree with the reviewer that these assumptions are specific to LDA (and some related methods). We added a remark clarifying that the named assumptions are not general for all topic models but specific to LDA.

6. *iv) Line 30: LDA term is introduced without having used before and explained.*

   We consent with the reviewer that acronyms should be introduced before they are used in the text. For the first occurrence we changed LDA to latent Dirichlet allocation (LDA)" and also provided references to the original paper as well as a text book (Anandarajan 2019) covering LDA. We also checked other acronyms used in text for being defined when introduced first.

7. *v) Paragraph starting on Line 32: It is somewhat unclear if you use or develop a new approach here. This should be made much more clear. Also relates to my main comment. If you mostly use, but in a new way, existing methods, I think much of the math and explanations in Sections 1.1, and 1.3 could be removed. Would rather focus on the intuition and what various input and output matrices(vectors) vectors actually represent. This is somewhat unclear to me.*

   Doing the revision, we tried to straighten out that the methodological contribution is centered around the way in which we combine existing methods to perform a task for which they have not been used before, i.e. topic modeling.

8. *vi) How do you handle the potential forward looking bias arising from estimating the textual model/clusters on the same data as you do the Granger causality tests? This should be addressed, and I do not think you comment on it or address it now, or?*

   We agree with the referee that this is a valid concern. Given that the structure of the PVTM topic model is obtained using the complete corpus, this structure might be subject to some implicit forward bias. However, the information entering the Granger causality tests is based only on past observations of topic weights and Google trend indices. To avoid the implicit forward bias stemming from the way the structure is obtained, we are currently considering a recursive estimation strategy similar to Kelly et al (2018), which is not part of this paper though. We have included a paragraph in the concluding section to raise awareness of this issue.

9. *vii) Line 76: Doc2Vec is mentioned, but not explained. Related to the point above and the articles methodological contribution.*

   We thank the referee for this comment. We agree that Doc2Vec could be better explained. We therefore added a paragraph at the end of the first section which details how exactly the single parts of PVTM work together and, in particular, the role of Doc2Vec.

10. *viii) The skipgram model. Is it not easier to just say that this is a logistic regression with latent parameters (word embeddings) and explanatory variables (context vectors)? Solved using a simple two-layer neural network?*

    We agree with the referee that this would be a very intuitive way to describe Skip-Gram. We thus added a paragraph to the Skip-Gram description in which we point to this interpretation.

11. *ix) Do you apply the standard negative sampling scheme when solving the model?*

    We thank the reviewer for pointing out the lacking information on the implementation of the model. In fact, we used the standard negative

sampling scheme making use of 5 negatives for each document-word pair. We have added this information in Subsection 2.2 describing the parameter settings.

12. *x) In Section 1.3, it is not exactly clear to me what the final output from all this is. Should perhaps give the reader some more intuition for this.*

    We are indebted to the referee for pointing this out. We have added a paragraph at the end of Section 1.3 which details the outputs and the intuition behind PVTM. Furthermore, the correct Figures 3 - 5 (and the additional ones in the Appendix) should also improve the understanding of the results obtained.

13. *xi) Below equation 11. Correct the text. Error in formatting.*

    Thank you for pointing out the formatting error, which has been fixed in the revised version.

14. *xii) Section2.1. I think you should explain better why this corpus is a good choice in the current setting (and potential extensions etc.)*

    We agree with the reviewer that the choice of data should be justified better. Therefore, we added a paragraph to Subsection 2.1 which details why we think the corpus is useful for identifying topics related to innovation and describing the diffusion process. We also mention limitations of this corpus and possible extensions for future research. Specifically, we added the following phrases: "We choose this corpus for the following reasons. First, because the corpus has a clear technology reference, which makes the identification of technology-related topics easier. Second, the corpus is available over a long period of time, 20 years, making it possible to measure long-term processes such as the diffusion of innovations in the first place. Third, newspaper articles are available in a timely manner, for example in comparison to patent data, so that adjustments or trends can be identified in the short term, which is an important criterion for Science, Technology and

Innovation (STI) policy One of the potential disadvantages is that the corpus is in German, which makes the results less accessible to non-German speaking readers. This disadvantage is partially mitigated by the fact that many of the frequently used terms in current technologies spring from English and can therefore be understood by non-German speaking readers. Apart from that, our question is specifically aimed at measuring the diffusion of innovations via newspaper articles in one country. Germany is an example of an industrialized country. Future studies could transfer these ideas to other countries."

15. *xiii) Line 191. K is used before it is defined and explained.*

    We thank the referee for this hint and we added details regarding K.

16. *xiv) I find Section 3 confusing. What is the point of this section? You start to talk about the results and figures, but then comes section 4. Likewise, if the innovations should follow the shape you illustrate, how can you test this? Should you test this?*

    We agree with the referee that the structure of the paper could be improved and decided to combine Sections 3 and 4. We also changed the heading of the new section to "Approaching the diffusion of innovations from related topics". Regarding whether the innovations follow the typical shape of diffusion of innovations shown in textbooks, we started working on a method to identify innovation related topics by their shape using functional data analysis, which, however, appears beyond the scope of the present paper.

17. *xv) In my version of the paper I could not find a figure of the word clouds that are mentioned in the text.*

    We have to apologize for our mistake when uploading the document and corresponding figures. Unfortunately, we have mixed up some files. Therefore, the submitted version did not include the wordclouds

in Figures 3 - 5. For the revision, we made sure to upload the correct images including the wordclouds.

18. *xvi) Line 311-318 feels somewhat speculative.*

    We thank the reviewer for this comment. We rephrased the text indicating more clearly that this statement is rather speculative.

19. *xvii) I do not understand why you need to use Johansen Trace Tests for cointegration in this setting. Would it not be enough to just difference the data to get them roughly stationary, and then run a battery of predictive tests (for many innovation terms)?*

    The referee makes a valid point arguing that applying Johansens cointegration framework might be exaggerated given that, on the one side, it is well known to lack robustness if certain assumptions are not satisfied and, on the other side, we do not know much yet about the time series properties of the data used for the Granger causality analysis. Therefore, we decided to follow his suggestions and rely on a more robust approach by considering the differenced data, which according to the usual tests, might be considered as roughly stationary. Nevertheless, one should keep in mind that his is a conservative approach as level information is lost and, consequently, a Granger causality test becomes less likely to indicate significant dependence for a limited finite number of lags. As a robustness check, we run also the Granger causality tests in levels assuming the existence of cointegration, and, as expected, found stronger indications for Granger causality. Nevertheless, we report only the results for the first differences as we consider them as more robust findings.

Response to Reviewer 2

The article describes a novel approach to measuring innovation. This approach consists of a combination of some well-known methods. By considering this framework, tests were executed by using real data sets. Additionally, to analyze the results, the authors compared the proposed index of importance with Google trends index. The analysis seems to be well-founded, but some crucial issues have to be corrected, as follows.

We are indebted to the referee for the careful reading of our review paper and his/her overall positive assessment. We will describe in detail below how we reacted to the specific comments made while preparing our revised version.

1. *You mentioned LDA in line 30, but did not define what LDA means. It would be better to write "Latent Dirichlet Allocation (LDA) ". Additionally, I think that you could cite a book that describes this method.*

   We consent with the reviewer that acronyms should be introduced before they are used in the text. For the first occurrence we changed "LDA" to "latent Dirichlet allocation (LDA)" and also provided references to the original paper as well as a text book (Anandarajan 2019) covering LDA. We also checked other acronyms used in text for being defined when introduced first.

2. *In Skip-Gram subsection, you used two different symbols to mean v_c.*

   We thank the referee for finding this inconsistency of notation. We made changes to make sure that the v symbol stands for one-hot encoded words, while $\mathbf{v_c}$ is used speficially for the current context word.

3. *In Subsection 1.2, you named a method as Word2Vec-SG. However, it is not clear the meaning of SG. Is it Skip-gram? Please, define it in the text.*

   The reviewer correctly points out that the meaning of Word2Vec-SG was not properly defined before using it. We added a short explanation to ensure that Word2Vec-SG is properly defined when using it.

4. *There is a problem with a citation on the least line of Subsection 1.2.*

We have fixed the error in the formatting of the citation.

5. *In subsection 2.2, you used a variable K that was not defined before.*

   We are indebted to the reviewer for pointing out that the meaning of K has not been defined before using it. We made the required change and defined K as the optimal number of Gaussian mixture components when introducing it.

6. *When you use BIC, do you mean Bayesian Information Criterion? It is not clear in the text.*

   We agree with the referee that BIC was not properly defined in the submitted version, which was due to formatting errors at the end of subsection 2.1. We have now fixed the formatting error and therefore BIC is well defined as Bayesian Information Criterion.

7. *Google Trends is crucial to your analysis. SO, I believe that you shall write a sentence explaining the index in the introduction. Furthermore, on page 8, you added a footnote with the website address, but this footnote should be on the first page where you mention it for the first time.*

   We thank the reviewer for this comment and agree that Google Trends is a crucial component to our analysis as it serves as a benchmark for the results obtained by the PVTM approach. Therefore, we added a paragraph to the Introduction that introduces Google Trends and also placed the url there.

8. *You mentioned that there are wordclouds in Figures 3 to 5, but there are no such figures in the PDF document.*

   We have to apologize for our mistake when uploading the document and corresponding figures. Unfortunately, we have mixed up some files. Therefore, the submitted version did not include the wordclouds in Figures 3 - 5. For the revision, we made sure to upload the correct images including the wordclouds.

9. *Figure 6 is not necessary because its information is already shown in Figures 3 to 5.*

   Again, we have to apologize for including the wrong files and included the correct image files now. This issue should thereby be resolved, as the content of Figure 6 is now different from the content shown in Figures 3-5.

10. *You described some well-known methods of your paper but did not explain about the Granger Causality Test (GCT). I think that it would be appropriate to describe GCT and the advantages of the method when employed as a forecast of a series. Furthermore, you shall better explain all the techniques that take part in the comparison between PVTM and GTI.*

    We agree with the referee that depending on the background of readers, the concept of Granger causality might not be well known. Therefore, we added some remarks regarding the intuitive interpretation of the Granger causality test. In principle, it measures whether adding past information from another indicator improves the prediction for an indicator of interest beyond the information already contained in the past development of that indicator, i.e. whether it contains additional information. For our application, the news ticker data is based on journalists comments about new technology, while Google trends rather reflect increasing interest on the side of consumers (what might be labelled intensive margin of innovation). Therefore, we expect that our novel indicator might be a leading indicator for Google trends and other diffusion measures, which can be tested by the Granger causality test.

11. *In lines 292 and 298, you wrote GT instead of GTI.*

    We agree with the referee that we mistyped GTI for GT in the mentioned lines and have altered the text accordingly.

12. *The results regarding Table 1 could be better explained.*

We decided to follow the suggestion of another referee to conduct the Granger causality testing in first differences as a kind of robust approach. Therefore, the results of cointegration testing (originally in Table 1) are not provided any more. We still conducted cointegration tests and also performed the Granger causality tests for the level variables as a robustness check, which confirmed the qualitative findings. However, given the focus on the more robust approach, these results have been removed from the paper.

13. *The data underlying the results are available on a website written in German. So, it was not possible to check it.*

    We have uploaded a script to github gist (`https://gist.github.com/davidlenz/2f14b28ead67ee1522c2d5fcbf76a45e`) with which the texts can be scraped from the website. As we do have explicit permission to work with the data but are not allowed to share the data, this is unfortunately the farthest we can go in providing access to our data source.

Response to Reviewer 3

The paper delivers a good contribution to the literature on applying the "Paragraph Vector Topic Model"(PVTM) to a data set or corpus of technolgy news articles. The PVT model is taken from the machine learning literature and adapted to the data set. The presentation of the PVTM is concise and clear.

We are indebted to the referee for the careful reading of our review paper and his/her overall positive assessment. We will describe in detail below how we reacted to the specific comments made while preparing our revised version.

1. *To evaluate the information content of the extracted topics, a Granger causality test is performed. All data are found to be I(1), the Johansen test confirms cointegration. The author proceeds using the Toda-Yamamoto approach by referring to Ltkepohl's textbook. This is inappropriate. This approach was developed for the case where the degree of integration is not clearly esdtablished. For the case here, the "trick" of adding an extra lag and estimating the VAR in levels is inferior to estimating a error correction model and analysing short-term and long-term aspects of cointegration separately (see the textbook on time series econometrics by Wolters, Kichgaessner and Hassler in this issue) by checking weak exogeneity and F-Tests/ Wald tests. Another appropriate test could also be found in the frequency domain Granger causality test proposed by Breitung and Candelon or Breitung and Schreiber.*

   We are indebted to the referee for pointing out alternative options for implementing the Granger causality test in a cointegration framework. Given the sensitivity of Johansens framework and a suggestion by another referee, we decided to concentrate on a model in first differences assuming that these are stationary (confirmed by standard unit-root testing). Obviously, we might lose some level information by doing so. Therefore, the findings from the Granger causality test for first differences is considered as a conservative estimator. Nevertheless, we also conducted the test in levels as a robustness check. Thereby, we considered two alternatives, the original test proposed by Granger and Lin

(1995) and the test proposed by Ltkepohl assuming that the level of (co-)integration is not clearly established. We agree that the original results presented in Table 1 appeared rather clear. However, extending the analysis to further topics the picture becomes a bit more mixed. Furthermore, the actual shape of the time series under consideration might not satisfy the main assumptions for Johansens proceeding. We mention that the results from these tests confirm the qualitative findings of the analysis in first differences.

2. *I recommend to redraft the section using an appropriate test approach.*

   Given the focus on the Granger causality test in first differences, the section has been redrafted.

Response to Reviewer 4

This paper uses PVTM to look at the diffusion of innovation using a corpus of articles from a German newspaper. The dynamics of each topic is then shown to carry some information which are different from what is found in the more standard measure using Google Trends. The paper is very well written and pedagogic and the analysis is done carefully. I mostly have minor comments. I would like to state again that I really liked the paper as it is, my main point regards the literature review that could be extended given the dynamics of this Field. But most of my other comments are minor and I would be happy to just see a discussion about them.

We are indebted to the referee for the careful reading of our review paper and his/her overall positive assessment. We will describe in detail below how we reacted to the specific comments made while preparing our revised version.

1. *Given the trans-disciplinary nature of the review, I believe it would be very insightful to have a short discussion of recent contributions regarding NLP applied to innovation related corpus (patents, scientic articles, newspaper, website etc...) and a brief overview of the evolution of the field (including some of your own works).*

   We thank the reviewer for this comment. We agree that a more comprehensive discussion of recent contributions would be insightful and have added such a discussion to the Introduction.

2. *In particular, how does your method compare to alternative (and older) classification methods (just to name a few examples: Nichols, 2014 using LDA, Chavalarias and Cointet, 2013, Bergeaud et al., 2017 using clustering and tdf/idf approaches and other older work e.g. Yoon and Park (2004) and Choi and Hwang, 2014) or more recent contribution (Abood and Feltenberger, 2018, Helmers et al., 2019)*

   We thank the referee for pointing out these references, which we read carefully and incorporated in the literature review.

3. *The number of words per article seems very low (278 on average). Is that specific*

tothe German language (with whom I am unfortunately not familiar) or is it specific to the type of news you are considering?

We thank the reviewer for this comment. In fact, the number of words of the news ticker articles seems to be in the range of what can be found in other applications. For comparison, we evaluated the number of words for the 20 Newsgroup training dataset and found that the average article in this corpus consists of 186 words, with a standard deviation of 523 words. We therefore consider the number of words in the heise news ticker articles to be of comparable length to news outlets in English. Thus, it does not appear to be an issue related to German.

4. *You picked 65% and 0.5% as thresholds in the data prepossessing. Could these thresholds be optimized in order to maximize one feature of your network (modularity, number of topics etc...)? More generally, can you discuss the sensitivity of your approach to adjusting these numbers?*

   We thank the reviewer for this comment on the choice of parameters. The lower threshold of 0.05% and the upper threshold of 65% are both lower compared to what is typically found in the literature (5% and 95%). For assessing the potential impact of alternative parameter choices, we evaluated three other thresholds (0.000005,0.65), (0.005, 0.65), (0.0005,1) and compared them to the original settings keeping other parameters (in particular the number of topics) constant. We found that changing the upper threshold has not any sensible impact on the results. By contrast, changing the lower threshold is more sensible. In particular using lower values (more liberal thresholds) might prevent finding some of the topics. This is due to the way the topic words are determined: The most similar words to a topic vector are used as topic words. When the lower threshold is very low, very rare words, which might even appear in a single document only, will be mapped close to the vector of the document in which they appeared.

Thus, with no restriction on the minimum frequency of appearance of a word, words that appear very few times and are very specific happen to be close to the topic vector and, consequently, are selected as topic words. We included a short discussion of these findings in the data preprocessing section.

5. *It would be super nice if you could validate the fact that your outcome is a good measure of the diffusion of innovation using an alternative dataset that is aiming at directly measuring such a diffusion. I am aware this is an impossible task for every topic, but perhaps you could find one or two nice examples. An example of such database is Comin and Hobijn (2009) but the world bank and the OECD also entertain cross country measures of innovation diffusion.*

   Thank you for the valuable suggestion. Unfortunately, the data used in Comin and Hobijin (2009) goes up only to 2001, which leaves a small overlap with our sample (1997 - 2016). Therefore, we tried to find alternative datasets which might be used for our specific comparison but did not find well suited resources yet. However, at least for one of our topics (wikipedia), we can use the number of new Wikipedians in Germany per month (available from `https://stats.wikimedia.org/DE/TablesWikipediaDE.htm`), which might be considered as an alternative indicator of the actual diffusion of Wikipedia. We compared this indicator with the PVTM importance measure for Wikipedia and added the figure to the appendix. In future work, we will search for further alternative data resources, which might be used to support the validity of the indicators constructed from our PVTM implementation.

6. *Why do you think your indicator carry additional information than the Google-Trends? My guess from the graph you shown is that the google trends reflects the intensive margin of innovation while the topic based measure you use refects the density this diffusion (people continue to search for information regarding tablets even when this technology is fully difused, but newspaper articles won't talk about it that much).*

Thank you for this valuable input. In fact, we might assume that the two indicators contain different information. The news ticker data is based on journalists comments about new technology, while Google trends rather reflect increasing interest on the side of consumers (what might be labelled intensive margin of innovation as you suggested). Therefore, we expect that our novel indicator might be a leading indicator for Google trends and other diffusion measures. This is tested by means of the Granger causality test, which intuitively measures the extent to which past information of one indicator contributes to forecasting the other one in addition to considering its own past. If our assumption is justified, we should find Granger causality running from our topic weight time series to the Google trend indicators.

7. *I find it interesting that contrary to the google trend measure that show spike corresponding to the release of new vintages, your measure is much smoother.*

   We thank the reviewer for this comment. We used the (one-sided) 12-period exponentially moving average for the Figures (not for the analysis though, where we used the original PVTM measures). We added this information to the caption of Figure 6 by adding "The PVTM measures are smoothed using the 12-period exponentially weighted moving average of the topic importance over time. We found statistical evidence that the unsmoothed PVTM topic importance measures Granger cause GTI for each of the presented topics."

8. *There are typos at the very end of Section 1*

   We have fixed the formatting errors at the end of Section 1.
