# Supplementary material for: Measuring the diffusion of innovations with paragraph vector topic models
Source: PLoS One. 2020 Jan 22;15(1):e0226685. doi: 10.1371/journal.pone.0226685 (PMC6976149; doi:10.1371/journal.pone.0226685)
Supplement: Attachment — Submitted filename: Response to Reviewers.pdf [file pone.0226685.s008.pdf]

Response to the Editor

1. *Please ensure that your manuscript meets PLOS ONE's style requirements, including those for file naming.*

   We carefully read the style requirements and did our best to satisfy all requirements.

2. *1. If the authors have adequately addressed your comments raised in a previous round of review and you feel that this manuscript is now acceptable for publication, you may indicate that here to bypass the "Comments to the Author" section, enter your conflict of interest statement in the "Confidential to Editor" section, and submit your "Accept" recommendation.*

   Reviewer 1: (No Response)

   Reviewer 2: All comments have been addressed

   Reviewer 3: All comments have been addressed

   Reviewer 4: All comments have been addressed

   -

3. *Is the manuscript technically sound, and do the data support the conclusions?*

   The manuscript must describe a technically sound piece of scientific research with data that supports the conclusions. Experiments must have been conducted rigorously, with appropriate controls, replication, and sample sizes. The conclusions must be drawn appropriately based on the data presented.

   Reviewer 1: Yes

   Reviewer 2: Yes

   Reviewer 3: Yes

   Reviewer 4: Yes

   -

4. *Has the statistical analysis been performed appropriately and rigorously?*

   Reviewer 1: Yes

   Reviewer 2: Yes

   Reviewer 3: Yes

   Reviewer 4: Yes

   -

5. *Have the authors made all data underlying the findings in their manuscript fully available?*

   The PLOS Data policy requires authors to make all data underlying the findings described in their manuscript fully available without restriction, with rare exception (please refer to the Data Availability Statement in the manuscript PDF file). The data should be provided as part of the manuscript or its supporting information, or deposited to a public repository. For example, in addition to summary statistics, the data points behind means, medians and variance measures should be available. If there are restrictions on publicly sharing data—e.g. participant privacy or use of data from a third party—those must be specified.

   Reviewer 1: Yes

   Reviewer 2: Yes

   Reviewer 3: Yes

   Reviewer 4: Yes

   -

6. *Is the manuscript presented in an intelligible fashion and written in standard English? PLOS ONE does not copyedit accepted manuscripts, so the language in submitted articles must be clear, correct, and unambiguous. Any typographical or grammatical errors should be corrected at revision, so please note any specific errors here.*

   Reviewer 1: Yes

Reviewer 2: Yes

Reviewer 3: Yes

Reviewer 4: Yes

-

Response to Reviewer 1

I think the authors have adequately addressed my comments raised in the previous round.

I only have two minor comments left.

We are again indebted to the referee for the careful reading of our review paper and his/her overall positive assessment. We will describe in detail below how we reacted to the specific comments made while preparing our revised version.

1. *There are still some typos in the text. I encourage the authors to fix these before publication. E.g., line 122*

   We thank the referee for this comment. We have again proofread the paper very carefully and are confident that no typos are left at this stage.

2. *The authors should check the reference list. Some of the articles listed as working papers are now published. E.g., reference [17]: Vegard H. Larsen, Leif A. Thorsrud, The value of news for economic developments, Journal of Econometrics, Volume 210, Issue 1, 2019, Pages 203-218.*

   We thank the referee for pointing out that some articles have been published in the meantime. We checked all unpublished paper and corrected the citation where appropiate.

Response to Reviewer 2

All the comments have been addressed in the new version. However, there is no label on the y-axis of the new figures.

We are again indebted to the referee for the careful reading of our review paper and his/her overall positive assessment. We will describe in detail below how we reacted to the specific comments made while preparing our revised version.

1. *There is no label on the y-axis of the new figures.*

   We consent with the reviewer that y-axis should have labels and therefore labeled the y-axis in the relevant figures with "PVTM Topic Importance".

Response to Reviewer 3

The authors reacted to the recommendations appropriately. Specifically, a robustness check of the Granger non-causality tests was integrated. The paper adds to the literature sufficiently. The method is very interesting and appropriate for the purpose of the study. Congratulations.

We are again indebted to the referee for the careful reading of our review paper and his/her overall positive assessment.

Response to Reviewer 4

Dear authors,

Thank you for your response. I am now satisfied with the current version of the revisions and the responses to my comments.

We are again indebted to the referee for the careful reading of our review paper and his/her overall positive assessment.
